# Right hemispheric structural connectivity and poststroke language recovery

**DOI:** 10.1002/hbm.26252

**Published:** 2023-02-28

**Authors:** Aleksi J. Sihvonen, Veronika Vadinova, Kimberley L. Garden, Marcus Meinzer, Tracy Roxbury, Kate O'Brien, David Copland, Katie L. McMahon, Sonia L. E. Brownsett

**Affiliations:** ^1^ Queensland Aphasia Research Centre University of Queensland Herston Australia; ^2^ School of Health and Rehabilitation Sciences University of Queensland Brisbane Australia; ^3^ Centre of Research Excellence in Aphasia Recovery and Rehabilitation La Trobe University Melbourne Australia; ^4^ Cognitive Brain Research Unit (CBRU) University of Helsinki Helsinki Finland; ^5^ Centre of Excellence in Music, Mind, Body and Brain University of Helsinki Helsinki Finland; ^6^ Department of Neurology University Medicine Greifswald Greifswald Germany; ^7^ School of Clinical Sciences, Centre for Biomedical Technologies Queensland University of Technology Brisbane Australia

**Keywords:** aphasia, language recovery, right hemisphere, stroke, structural connectometry

## Abstract

Poststroke aphasia typically results from brain damage to the left‐lateralized language network. The contribution of the right‐lateralized homologues in aphasia recovery remains equivocal. In this longitudinal observational study, we specifically investigated the role of right hemisphere structural connectome in aphasia recovery. Twenty‐two patients with aphasia after a left hemispheric stroke underwent comprehensive language assessment at the early subacute and chronic stages. A novel structural connectometry approach, using multi‐shell diffusion‐weighted MRI data collected at the early subacute stage, was used to evaluate the relationship between right hemisphere white matter connectome and language production and comprehension abilities at early subacute stage. Moreover, we evaluated the relationship between early subacute right hemisphere white matter connectome and longitudinal change in language production and comprehension abilities. All results were corrected for multiple comparisons. Connectometry analyses revealed negative associations between early subacute stage right hemisphere structural connectivity and language production, both cross‐sectionally and longitudinally (*p*_FDR_ < .0125). In turn, only positive associations between right hemisphere structural connectivity and language comprehension were observed, both cross‐sectionally and longitudinally (*p*_FDR_ < .0125). Interhemispheric connectivity was highly associated with comprehension scores. Our results shed light on the discordant interpretations of previous findings, by providing evidence that while some right hemisphere white matter pathways may make a maladaptive contribution to the recovery of language, other pathways support the recovery of language, especially comprehension abilities.

## Graphical abstract

The contribution of the right‐lateralized homologues in aphasia recovery remains equivocal. Here, we provide evidence that while some right hemisphere white matter pathways may make a maladaptive contribution to the recovery of language, other pathways support the recovery of language, especially comprehension abilities.

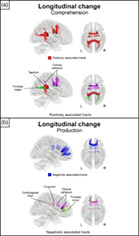

## 1 INTRODUCTION

Aphasia is a common and debilitating consequence of left hemisphere stroke, reducing health‐related quality of life more than both cancer and dementia (Lam & Wodchis, 2010). Despite treatment efforts, up to 60% of the patients remain aphasic into the chronic phase (Pedersen et al., 1995). While recovery can continue into the chronic phase of recovery (Fleming et al., 2020), the steepest recovery trajectory typically occurs within 2–6 weeks of stroke (Pedersen et al., 1995). This reflects the importance of the spared neural structures for aphasia recovery. Identifying structures that contribute to the recovery from aphasia is essential if clinicians are to be able to provide reliable prognoses and individualize treatment strategies to optimize recovery.

A body of research has shown that greater lesion load in the left arcuate fasciculus predicts poor language outcome in aphasia (Alyahya et al., 2020; Fridriksson et al., 2013; Hillis et al., 2018; Marchina et al., 2011). However, using hierarchical regression analyses, while greater lesion volume in the acute stage emerged as an independent predictor of more severe chronic aphasia, lower left arcuate fasciculus volume did not (Forkel et al., 2014). In contrast, greater right arcuate fasciculus volume in the acute stage has been shown to be an independent predictor of less severe chronic aphasia, together with age, sex, and lesion size, explaining 57% of the variance in chronic poststroke language outcomes (Forkel et al., 2014). Recent aphasia studies have also identified white matter (WM) tracts outside the homologous language pathways, including right cingulum and cortico‐subcortical projection pathways as well as corpus callosum, positively predicting verb retrieval (Dresang et al., 2021), semantic and phonological processing abilities (Hula et al., 2020), and speech fluency (Pani et al., 2016) in aphasia. Moreover, volume within contralesional frontotemporal WM structures has been shown to predict treatment response to a high dose auditory comprehension therapy (Fleming et al., 2020). Together, these studies suggest that structural connectivity in the hemisphere contralateral to the lesion plays an important role in recovery of aphasia.

However, the details regarding the relationship between right hemisphere structural connectivity and aphasia recovery remain unclear. First, conflicting with previous findings (Forkel et al., 2014), recent evidence suggests that higher mean fractional anisotropy (FA) in the right arcuate fasciculus is associated with poorer naming recovery (Keser et al., 2020). Moreover, better semantic and phonological processing abilities in aphasia have shown both negative and positive associations with quantitative anisotropy (QA) values, even within the same WM tracts (Hula et al., 2020). Second, studies have largely focused on one or two WM tracts (Forkel et al., 2014; Keser et al., 2020), analyzed regional FA (Pani et al., 2016) or mean FA representing the whole WM tract (Keser et al., 2020), or focused on selective single‐word language outcomes, such as naming (Keser et al., 2020) and fluency (Pani et al., 2016). Third, to our knowledge, there are no diffusion MRI (dMRI) studies focusing on predicting comprehensive language outcomes, that is, both production and comprehension, after stroke in relation to the right hemisphere WM structure. Comprehensive assessment of outcomes is important for targeted rehabilitation planning and accounts for different aspects of language that can have different recovery trajectories. As suggested earlier (Keser et al., 2020), the role of the right hemispheric WM structure in aphasia recovery might be heterogenous and highly related to specific language function and anatomical structures.

Here, we specifically assessed if right hemisphere WM structural connectivity was associated with spoken language production and comprehension in patients with aphasia following left hemisphere stroke. To do so, we utilized right hemisphere connectometry (Yeh, Badre, et al., 2016) based on multi‐shell dMRI data acquired at the early subacute stage and language production and comprehension assessments at both the early subacute (2–6 weeks) and chronic (>6 months) poststroke stages (Table 1). Connectometry is a novel dMRI analytic approach that utilizes QA‐based permutation testing to identify WM tracts associated with a variable of interest. QA is a diffusion orientation distribution function (ODF)‐based measure for resolving multiple fibers that improves fiber tracking. Due to there being less susceptibility to the partial volume effects of free water, crossing fibers, and nondiffusive materials, this approach has been shown to be more sensitive than conventional voxel‐based or track‐based analyses utilizing FA (Jin et al., 2019; Yeh et al., 2013). QA‐based connectometry has recently been used to uncover WM tracts supporting word production (Hula et al., 2020) and verb retrieval (Dresang et al., 2021) in aphasia. Based on the previous dMRI evidence (Forkel et al., 2014; Keser et al., 2020; Kourtidou et al., 2021; Pani et al., 2016), and data suggesting that the corpus callosum mediates speech perception in the brain (Steinmann & Mulert, 2012), we hypothesized that (i) language production and comprehension outcomes would be associated with greater QA in the corpus callosum, (ii) language production outcomes would be associated with both negative and positive QA, and (iii) language comprehension outcomes would be associated with different WM factors to production.

**TABLE 1:** Patient characteristics/demographics in both the early subacute and chronic stages.

	Early subacute	Chronic
Participants (*n*)	22	15
Age (mean ± SD years)	64.02 ± 11.16	61.33 ± 8.15
Males	16	11
ISCED level of education	4.14 ± 1.93	4.27 ± 1.87
Ischemic stroke (*n*)	18	13
Days poststroke to MRI (mean ± SD days)	30.05 ± 10.67	207.13 ± 18.46
Lesion volume (mL)	32.03 ± 25.96	
Speech production *t*‐score	54.3 (8.8)	60.7 (8.8)
Speech comprehension *t*‐score	53.6 (7.6)	56.4 (7.6)

Abbreviations: ISCED, International Standard Classification of Education; *n*, number of participants; SD, standard deviation.

## 2 MATERIALS AND METHODS

### 2.1 Participants and study design

This observational study is a part of the “Predicting and Promoting Aphasia Recovery” study approved by the Royal Brisbane and Women's Hospital Human Research Ethics Committee (HREC/16/QRBW/376). All participants gave informed written consent in accordance with the Declaration of Helsinki. Inclusion criteria were: first‐ever left hemisphere stroke with aphasia as measured by an Aphasia Quotient of <93.8 using the Western Aphasia Battery—Revised (Kertesz, 2007); availability for an initial assessment at 2–6 weeks poststroke onset; medically and cognitively able to participate in a research intervention; and English as a dominant language prior to their stroke. Exclusion criteria were: neurological disease or disorder other than the stroke; contraindications to MRI; severe dysarthria or apraxia of speech; severe auditory comprehension deficits prohibiting informed consent; or substance dependence within the 12 months prior to their stroke. Twenty‐two participants with a mean lesion volume of 32.03 mL (SD 25.96) were recruited from 10 hospital sites in the southeast Queensland region, Australia 2017–2021. Participant details and demographics are reported in Table 1.

### 2.2 Language assessment

To screen for aphasia prior to recruitment into the study, the Western Aphasia Battery—Revised was completed to obtain Aphasia Quotients (Kertesz, 2007). This was conducted at 2–6 weeks poststroke and within 2 weeks of the subacute brain imaging. Detailed language assessments at each time point included the Comprehensive Aphasia Test (CAT) (Swinburn et al., 2005). These were also collected within 24 h of imaging at the subacute stage. An overall score for spoken language comprehension was derived from the combination of raw scores from the auditory single word, sentence, and paragraph comprehension subtests of the CAT. An overall score for spoken language production was derived from the combination of raw scores from the object and verb naming, verbal fluency subtests of the CAT. All raw scores were then converted to *t*‐scores.

### 2.3 MRI data acquisition and reconstruction

All neuroimaging data were acquired on a 3‐Tesla Siemens Magnetom Prisma MRI scanner (Siemens, Erlangen, Germany) with a 20‐channel head/neck coil at the Herston Imaging Research Facility. For each participant, a high‐resolution structural 3D T1‐weighted image (MP2‐RAGE, O'Brien et al., 2014; TR = 4000 ms; TE = 2.91 ms; TI 1 = 700 ms; TI 2 = 2220 ms; voxel size = 1.0 × 1.0 × 1.0 mm^3^) and dMRI data covering the whole brain and brainstem were acquired (TE = 81 ms, TR = 4000 ms, 2.0 × 2.0 × 2.0 mm^3^ resolution, 11 images with *b* = 0 s/mm^2^, 20 directions at *b* = 1000 s/mm^2^, 60 directions at *b* = 3000 s/mm^2^).

Diffusion images were processed using FSL (www.fmrib.ox.ac.uk/fsl) and MRTrix (www.mrtrix.org). Images were denoised and corrected for distortion, eddy currents, and motion (Andersson & Sotiropoulos, 2016; Veraart et al., 2016). After this, the dMRI data were reconstructed in the Montreal Neurological Institute space using q‐space diffeomorphic reconstruction (Yeh & Tseng, 2011) that allows for the construction of spin distribution functions (Yeh et al., 2010) in DSI Studio (http://dsi-studio.labsolver.org, version April 7, 2021). During the reconstruction, a mask is used to filter out non‐WM structures, increasing the reconstruction efficacy. The b‐table was checked by an automatic quality control routine to ensure its accuracy (Schilling et al., 2019). Normalization was carried out using the anisotropy map of each participant and a diffusion sampling length ratio of 1.25. Quality of the normalization was inspected using the *R* ^2^ values denoting goodness of fit between the participant's anisotropy map and template. Each participant's forceps major and minor were inspected and used as an anatomical benchmark to confirm the normalization quality (Hula et al., 2020). The restricted diffusion was quantified using restricted diffusion imaging (Yeh et al., 2017), and QA was extracted as the local connectome fingerprint (Yeh, Vettel, et al., 2016) and used in the connectometry analysis.

### 2.4 Connectometry analysis

Diffusion MRI connectometry (Yeh, Badre, et al., 2016) analysis was carried out using DSI Studio. Connectometry is a reasonable new statistical method that includes mapping and analysis of local connectomes, that is, the degree of connectivity between adjacent voxels within a WM fascicle defined by the density of the diffusing spins. As a result, instead of mapping the entire end‐to‐end connectome, connectometry tracks only the segment of fiber bundle that exhibits significant association with the study variable, here language production and comprehension. To do this, dMRI data are reconstructed into a standard template space on to a local connectome matrix from the studied sample. Study‐relevant variables are then associated with this local connectome matrix to identify local connectomes expressing significant associations with the variable of interest. Using ODF‐based measure (QA) for resolving multiple fibers, these local connectomes are then tracked along the core pathway of a fiber bundle using a fiber tracking algorithm within a tractography atlas and compared with a null distribution of coherent associations using permutation statistics. In summary, connectometry analyzes significant QA associations with a variable of interest or QA differences between two groups along the pathways themselves as compared to mean FA in a voxel or representing a whole tract. As the dMRI data are reconstructed into standard space and tracking is based on template, it also minimizes bias induced by manual tracking. The minimum length is set by voxel threshold (here 30 voxels). While the default analysis metric is QA, connectometry allows for measures such as FA.

Four multiple regression models were used to identify the early subacute stage right hemisphere local connectomes associated with (i) comprehension and (ii) production at early subacute stage cross‐sectionally. Moreover, early subacute stage local connectomes were used to predict longitudinal (chronic > early subacute) change in (iii) production and (iv) comprehension scores. Lesion volume and age were included as covariates in all models. Local connectomes with *t*‐scores exceeding 3 were selected (Hula et al., 2020) and tracked using a deterministic fiber tracking algorithm (Yeh et al., 2013) to obtain correlational tractography. The tracks were filtered by topology‐informed pruning (Yeh et al., 2019) with 8 iterations, and a length threshold of 30 voxel distance was used to identify significant tracts. Bootstrap resampling with 4000 randomized permutations was used to obtain the null distribution of the track length and estimate the false discovery rates (FDRs) to correct for multiple comparisons in all four regression analyses. Furthermore, due to four calculated regression models, alpha level was set to *p* < .0125 (Bonferroni correction). Data met the linear regression assumptions of normality and homoscedasticity, with an absence of multicollinearity.

## 3 RESULTS

Multi‐shell dMRI and language data were collected in the early subacute stage from the recruited 22 participants, 15 of which also had language data collected at the chronic stage. Data for seven participants were not collected at the chronic stage due to COVID‐19 restrictions (*n* = 2), lost to follow‐up (*n* = 1), withdrawal of participation (*n* = 2), or unsuitability for the word‐retrieval therapy (*n* = 2). There was no additional missing data.

Eight people with aphasia participated in an 8‐week word‐retrieval treatment delivered between the early subacute and chronic stages. To ensure that the treatment did not affect the current results, an independent samples *t*‐test of the data was calculated and showed no difference between usual care and treatment groups for either language comprehension or production scores, in either the early subacute or chronic stage (*p* = .399–.943; see Supplementary material).

While the analyses were restricted to right hemisphere tracts, including the interhemispheric tracts feeding into the lesioned left hemisphere, we calculated individual lesion loads in the interhemispheric tracts for the participants to rule out the artifact of left hemisphere lesions impacting both behavior and the integrity of these tracts. The mean lesion load in the interhemispheric tracts, that is, all parts of corpus callosum, was 0.3% (SD 0.35%) that could be considered very low. In addition, the lesion load in the interhemispheric tracts did not significantly correlate with the comprehension or production *t*‐scores at the early subacute stage or longitudinally (*p* = .120–.739).

### 3.1 Early subacute stage

**FIGURE 1:**
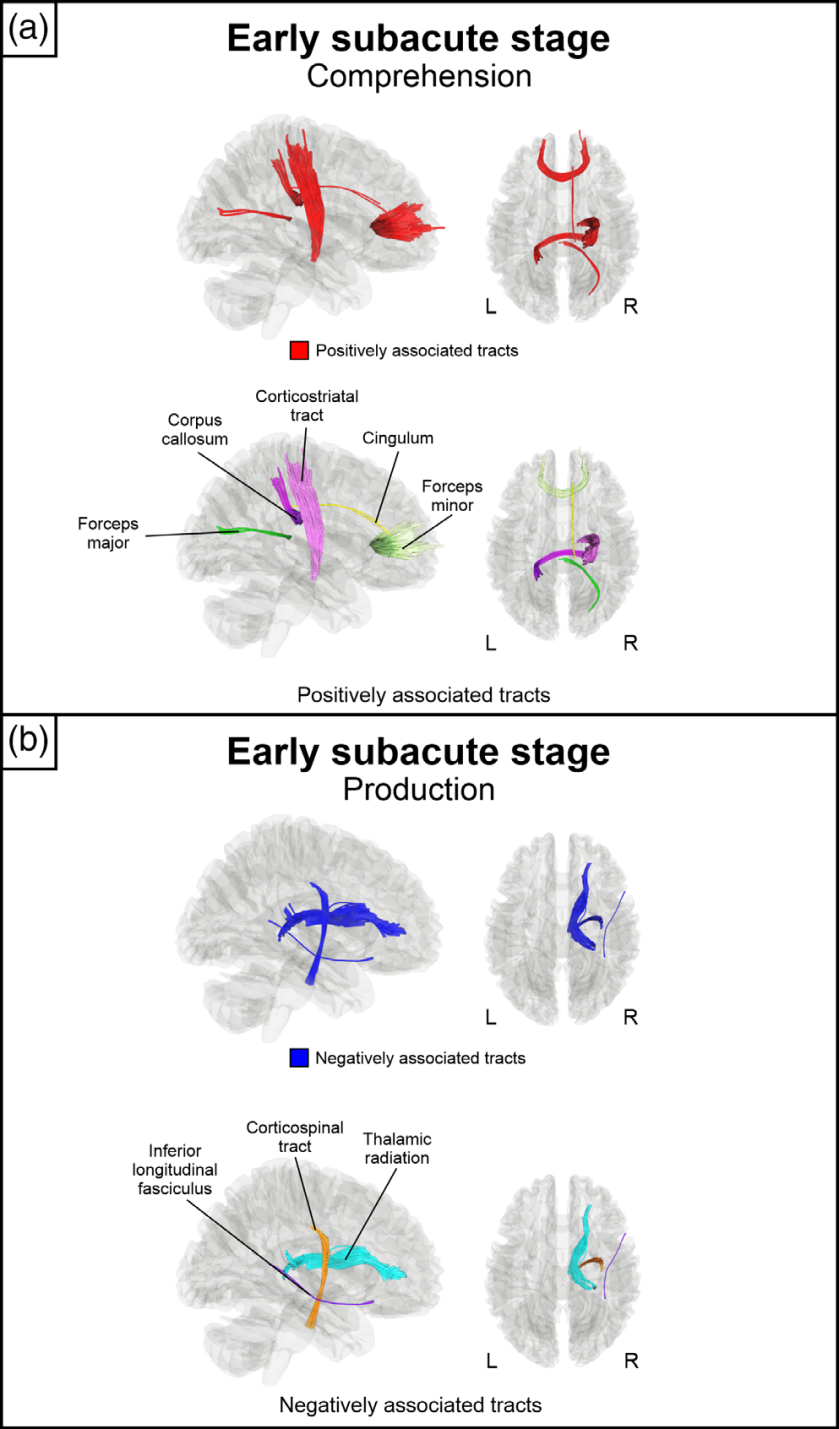
Early subacute stage connectomes associated positively and negatively with early subacute comprehension and production scores. (a) Positively associated tracts with comprehension. (b) Negatively associated tracts with production. All results are thresholded at FDR < .0125, *N* = 22. FDR, false discovery rate; L, left; R, right.

First, we evaluated which right hemisphere local connectomes were associated with language comprehension and production abilities at the early subacute stage cross‐sectionally (*n* = 22). Higher comprehension *t*‐scores at the early subacute stage were associated with greater QA in segments of the right corticostriatal tract and cingulum as well as the corpus callosum (body, forceps minor and forceps major) (Figure 1a) (*p*_FDR_ < .0125). Negative associations were not observed. Higher production *t*‐scores were associated with lower QA in segments of the right corticospinal tract, thalamic radiation, and inferior longitudinal fasciculus (Figure 1b) (*p*_FDR_ < .0125). Positive associations were not observed.

### 3.2 Longitudinal change in language scores

**FIGURE 2:**
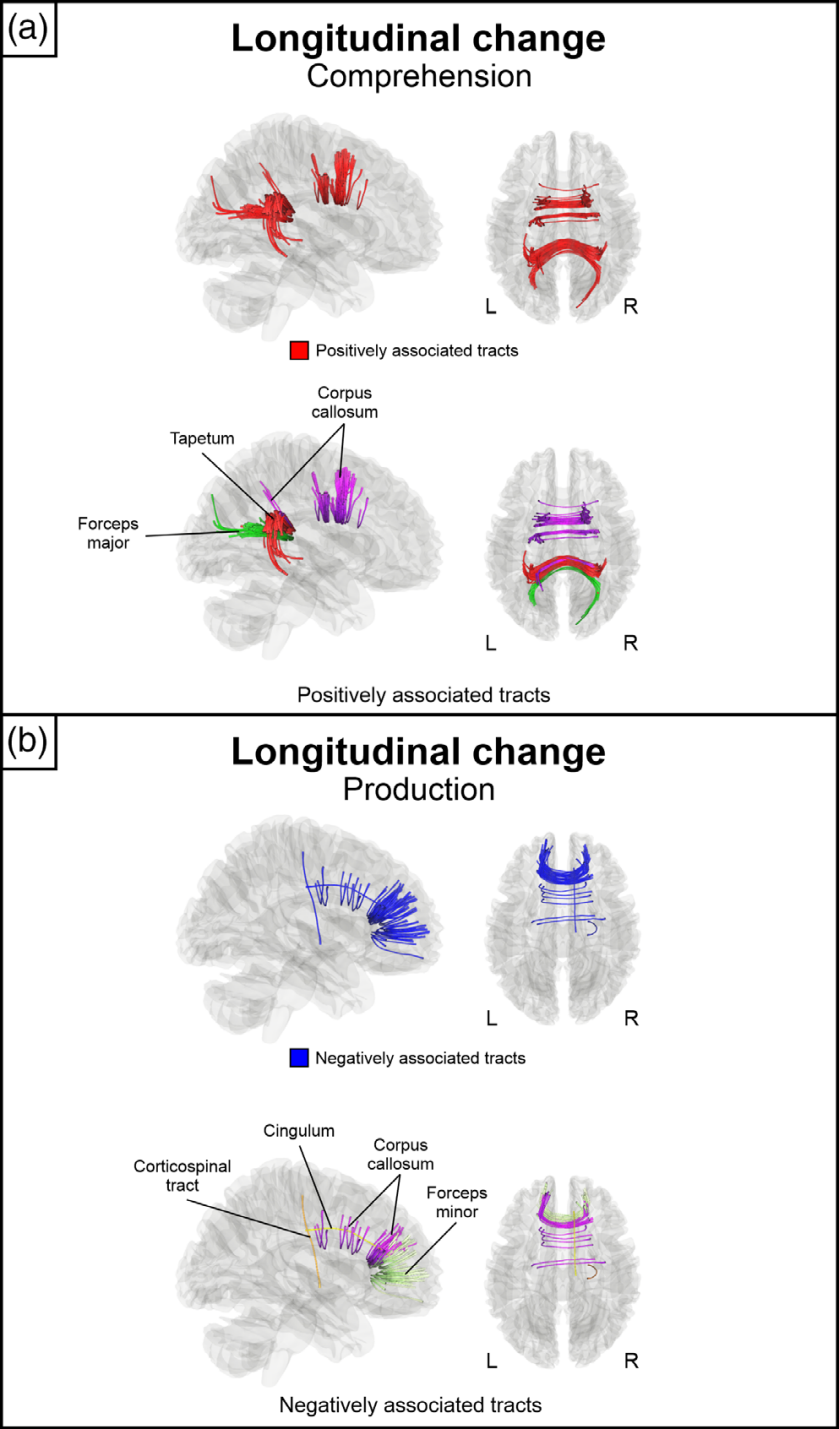
Significant associations between the early subacute stage connectomes and longitudinal change in comprehension and production scores. (a) Positively associated tracts with comprehension. (b) Negatively associated tracts with production. All results are thresholded at FDR < .0125, *N* = 15. FDR, false discovery rate; L, left; R, right.

Next, we evaluated which early subacute stage right hemisphere local connectomes were associated with longitudinal change (chronic > early subacute) in comprehension and production *t*‐scores (*n* = 15). Greater longitudinal improvement in language comprehension *t*‐scores was associated with greater QA in the corpus callosum (body, forceps major and tapetum) (Figure 2a) (*p*_FDR_ < .0125). Negative associations were not observed.

In language production, only significant negative associations were observed, showing greater longitudinal improvement in production *t*‐scores in association with lower QA in segments of right corticospinal tract and cingulum as well as the corpus callosum (body, forceps minor) (Figure 2b) (*p*_FDR_ < .0125).

To ensure that left hemisphere structural characteristics have been adequately accounted for in the analyses, we repeated the abovementioned longitudinal analyses and evaluated early subacute whole‐brain local connectome associations with longitudinal change (chronic > early subacute) in comprehension and production *t*‐scores (*n* = 15). Moreover, in addition to age and lesion volume, baseline performance was included as an additional covariate to account for possible confounding effects (Hope et al., 2019). The results remained largely unchanged; see the Supplementary material.

## 4 DISCUSSION

This study set out to evaluate the association between early subacute right hemisphere WM structural connectivity patterns and spoken language production and comprehension in patients with aphasia. We provide novel evidence that while some right WM pathways are associated with better aphasia outcomes, others are associated with poorer recovery. The nature of this association was dependent on both the aspect of language being considered (i.e., comprehension or production) and the stage of recovery. Compared to previous evidence derived on diffusion tensor imaging (DTI) (Forkel et al., 2014; Keser et al., 2020; Pani et al., 2016), we utilized generalized q‐sampling imaging (GQI) (Yeh et al., 2010), a model‐free method that enables reconstruction of crossing fibers more completely, consistently, and accurately (Jin et al., 2019). This QA‐based hodological approach that identifies local connectomes, that is, segments of WM tracts, expressing significant associations with study‐relevant variables has been shown to outperform traditional FA‐based analyses by being more specific to individual's connectivity patterns (Yeh, Vettel, et al., 2016) and less susceptible to the partial volume effect (Yeh et al., 2013), yielding more precise information of the connectome. Our results make an important contribution to current debates in the literature regarding the relationship between right hemisphere structural connectivity and the recovery of aphasia, and the importance of the spared WM neural structures for language, especially comprehension, and the recovery of language after stroke.

Our findings are the first to consider the contribution of all right hemisphere WM tracts across the recovery profile. Previous research has investigated these contributions cross‐sectionally, in the early subacute phase (Forkel et al., 2014) or in the chronic phase of recovery (Dresang et al., 2021; Fleming et al., 2020; Fridriksson et al., 2013; Hula et al., 2020; Kourtidou et al., 2021; Marchina et al., 2011). Keser et al. (2020) investigated the longitudinal contribution of two right hemisphere WM tracts to language recovery after stroke in 10 stroke patients. These authors found that greater mean FA values of the right arcuate fasciculus, a prominent dorsal tract interconnecting frontotemporal and parietal regions, were associated with poor recovery of naming. They interpreted this finding as a greater reliance on left hemisphere structures better supporting language recovery. Moreover, previous evidence has shown that intensive intonation‐based therapy induces reductions in regional FA values in the right inferior frontal gyrus in relation to improved language production (Wan et al., 2014). Paralleling these results, we observed only negative associations between the right hemisphere local connectomes and recovery of language production abilities. Both early subacute stage cross‐sectional and longitudinal analyses revealed significant tracts that connect to the right frontal lobe (anterior thalamic radiation and the anterior and middle part of the corpus callosum, respectively). Previous evidence has shown that thalamic radiations show rightward lateralization in patients with poststroke aphasia and that this pattern is associated with poorer naming (Keser et al., 2021). The association of higher QA in the anterior and middle parts of corpus callosum to longitudinal decrease in production abilities might reflect the functional reorganization and posterior shift of the right frontal cortical regions, supporting naming during the recovery of aphasia, such has been observed in noninvasive brain stimulation studies of aphasia (Harvey et al., 2017).

Forkel et al. (2014) have reported that acute stage right arcuate fasciculus volume was an independent predictor of less severe chronic aphasia at 6 months in a sample of 16 patients. However, this study neither evaluated the relationship between acute stage tract volume and longitudinal change in language scores nor found any significant correlation between measures of mean FA values in the three segments of the right arcuate fasciculus and aphasia severity at either time point. The more sensitive QA‐based analyses presented in this study revealed significant negative associations between language production outcomes in aphasia and right hemisphere WM structures but these did not include the arcuate fasciculus. While we did not include volumetric evaluation of the right hemispheric tracts in the current study, the previous results (Forkel et al., 2014) could reflect pre‐existing right hemisphere networks that may support functional compensation in aphasia. The degree of lateralization of arcuate fasciculus is heterogeneous, and bilateral representation might ultimately be advantageous for language (Catani et al., 2007).

While Keser et al. (2020) focused on naming tasks only, our study also included measures of language comprehension. Studies to date that have investigated the contribution of the right hemisphere's WM integrity to language recovery have focused primarily on single word naming (Keser et al., 2020) or fluency tasks (Pani et al., 2016), with only one study using a more functional measure of language production (Wan et al., 2014). Given that anomia is the most common residual deficit in aphasia (Hillis et al., 2018), naming tasks are frequently used in aphasia research. However, the linguistic processing demands involved in naming a high frequency item are different to those needed for understanding a spoken word.

In our study, the subdivisions of the corpus callosum were also differentially associated with production and comprehension outcomes. While the middle and posterior parts of the body as well as tapetum and forceps major were associated with better outcomes for comprehension of language; forceps minor was positively associated with comprehension outcomes at the early subacute stage and negatively associated with longitudinal production change. This discrepancy might relate to dynamic language reorganization that is highly dependent on the lesion location (Stockert et al., 2020); however, we are unable to make more fine‐grained analyses to interrogate this with our current sample. In general, these dissociations suggest that to understand the contribution of the right hemisphere in language recovery after stroke, both aspects of language must be considered. We are not aware of any other studies that have used dMRI data to look at longitudinal production and comprehension of language after stroke in relation to right hemisphere WM structure. Given that data from other imaging modalities have shown that language comprehension, both at the single word level (Klimovich‐Gray & Bozic, 2019) and the sentence level (Brownsett et al., 2014), engages bilateral domain‐general networks, in addition to traditional cortical areas associated with language, it is not surprising that the WM tracts supporting these tasks are likely to differ depending on the complexity of the stimuli presented. During auditory speech comprehension, the left and right hemisphere speech processing pathways interact reciprocally via the corpus callosum, integrating syntactic and prosodic features of speech (Sammler et al., 2010). This specificity of involvement of the corpus callosum was highlighted through the use of more complex measures of language that necessitate involvement of both domain‐specific and domain‐general neural networks, and the structural connections underpinning these networks. We suggest that by investigating comprehension tasks, in addition to more frequently employed production tasks, our analysis was able to uncover more widespread WM networks required to support everyday language skills.

By using a QA‐based connectometry approach, rather than more conventional regional FA (Pani et al., 2016) or mean FA across a whole WM tract (Keser et al., 2020), we were also able to demonstrate the contribution of both commissural and right hemispheric tracts that are associated with the recovery of language after stroke. Using this methodology, the discordant results observed in studies to date (Fridriksson et al., 2013; Keser et al., 2020; Marchina et al., 2011), which have used mean FA to investigate the contribution of a single tract associated with language production, may be explained by a combination of (a) the aspect of language being assessed, (b) the recovery phase being investigated, and (c) the task used to assess language. By adopting a connectometry approach, we have added to the growing evidence of the contribution of WM tracts outside the homologous language pathways to predicting better language outcome in aphasia (Dresang et al., 2021; Hula et al., 2020; Pani et al., 2016). It must be acknowledged that recovery among individuals with aphasia remains highly variable, depending on the site and volume of stroke as well as brain reserve and other variables (Kiran & Thompson, 2019), and therefore, given that the sample sizes remain modest in both the present and previously published studies focusing on right hemispheric structural connectivity in aphasia recovery (Forkel et al., 2014; Keser et al., 2020; Pani et al., 2016), future larger‐scale studies are required to assess the out‐of‐sample predictive value of the current results and enable generalization to the wider aphasic population. While it must be acknowledged that we are unable to determine the extent to which therapy proved to eight of the people with aphasia, contributed to the differential contribution of tracts to language production and comprehension scores, it seems unlikely that the amount of therapy received in this data set would have contributed to the changes, given there were no differences between groups on the behavioral scores measured at either time point.

## 5 CONCLUSION

In summary, the use of longitudinal data, more comprehensive measures of language, and QA‐based connectometry has provided novel evidence that structural connectivity plays an important role in recovery of language after stroke both within the hemisphere contralateral to the lesion, and between hemispheres. Together, these findings provide crucial information about the importance of the spared WM neural structures for aphasia recovery. Larger studies are needed to confirm our findings to enable clinicians to provide more specific prognoses for people living with aphasia.

## CONFLICT OF INTEREST STATEMENT

The authors report no conflict of interest.

## Supporting information


**Data S1:** Supporting information

## Data Availability

Anonymized data reported in this manuscript are available from the corresponding author upon reasonable request and subject to approval by the appropriate regulatory committees and officials.

## References

[hbm26252-bib-0001] Alyahya, R. S. W. , Halai, A. D. , Conroy, P. , & Lambon, M. A. (2020). A unified model of post‐stroke language deficits including discourse production and their neural correlates. Brain, 143(5), 1541–1554. 10.1093/brain/awaa074 32330940 PMC7241958

[hbm26252-bib-0002] Andersson, J. L. R. , & Sotiropoulos, S. N. (2016). An integrated approach to correction for off‐resonance effects and subject movement in diffusion MR imaging. NeuroImage, 125, 1063–1078. 10.1016/J.NEUROIMAGE.2015.10.019 26481672 PMC4692656

[hbm26252-bib-0003] Brownsett, S. L. E. , Warren, J. E. , Geranmayeh, F. , Woodhead, Z. , Leech, R. , & Wise, R. J. S. (2014). Cognitive control and its impact on recovery from aphasic stroke. Brain, 137(1), 242–254. 10.1093/brain/awt289 24163248 PMC3891442

[hbm26252-bib-0004] Catani, M. , Allin, M. P. G. , Husain, M. , Pugliese, L. , Mesulam, M. M. , Murray, R. M. , & Jones, D. K. (2007). Symmetries in human brain language pathways correlate with verbal recall. Proceedings of the National Academy of Sciences of the United States of America, 104(43), 17163–17168. 10.1073/pnas.0702116104 17939998 PMC2040413

[hbm26252-bib-0005] Dresang, H. C. , Hula, W. D. , Yeh, F.‐C. , Warren, T. , & Dickey, M. W. (2021). White‐matter neuroanatomical predictors of aphasic verb retrieval. Brain Connectivity, 11(4), 319–330. 10.1089/brain.2020.0921 33470167 PMC8112714

[hbm26252-bib-0006] Fleming, V. , Brownsett, S. , Krason, A. , Maegli, M. A. , Coley‐Fisher, H. , Ong, Y. H. , Nardo, D. , Leach, R. , Howard, D. , Robson, H. , Warburton, E. , Ashburner, J. , Price, C. J. , Crinion, J. T. , & Leff, A. P. (2020). Efficacy of spoken word comprehension therapy in patients with chronic aphasia: A cross‐over randomised controlled trial with structural imaging. Journal of Neurology, Neurosurgery and Psychiatry, 92(4), 418–424. 10.1136/jnnp-2020-324256 33154182 PMC7611712

[hbm26252-bib-0007] Forkel, S. J. , De Schotten, M. T. , Dell'Acqua, F. , Kalra, L. , Murphy, D. G. M. , Williams, S. C. R. , & Catani, M. (2014). Anatomical predictors of aphasia recovery: A tractography study of bilateral perisylvian language networks. Brain, 137(7), 2027–2039. 10.1093/brain/awu113 24951631

[hbm26252-bib-0008] Fridriksson, J. , Guo, D. , Fillmore, P. , Holland, A. , & Rorden, C. (2013). Damage to the anterior arcuate fasciculus predicts non‐fluent speech production in aphasia. Brain, 136(11), 3451–3460. 10.1093/brain/awt267 24131592 PMC3808690

[hbm26252-bib-0009] Harvey, D. Y. , Podell, J. , Turkeltaub, P. E. , Faseyitan, O. , Coslett, H. B. , & Hamilton, R. H. (2017). Functional reorganization of right prefrontal cortex underlies sustained naming improvements in chronic aphasia via repetitive transcranial magnetic stimulation. Cognitive and Behavioral Neurology, 30(4), 133–144. 10.1097/WNN.0000000000000141 29256908 PMC5797702

[hbm26252-bib-0010] Hillis, A. E. , Beh, Y. Y. , Sebastian, R. , Breining, B. , Tippett, D. C. , Wright, A. , Saxena, S. , Rorden, C. , Bonilha, L. , Basilakos, A. , Yourganov, G. , & Fridriksson, J. (2018). Predicting recovery in acute poststroke aphasia. Annals of Neurology, 83(3), 612–622. 10.1002/ana.25184 29451321 PMC5867273

[hbm26252-bib-0011] Hope, T. M. H. , Friston, K. , Price, C. J. , Leff, A. P. , Rotshtein, P. , & Bowman, H. (2019). Recovery after stroke: Not so proportional after all? Brain, 142(1), 15–22. 10.1093/brain/awy302 30535098 PMC6308308

[hbm26252-bib-0012] Hula, W. D. , Panesar, S. , Gravier, M. L. , Yeh, F. C. , Dresang, H. C. , Dickey, M. W. , & Fernandez‐Miranda, J. C. (2020). Structural white matter connectometry of word production in aphasia: An observational study. Brain, 143(8), 2532–2544. 10.1093/brain/awaa193 32705146 PMC7447522

[hbm26252-bib-0013] Jin, Z. , Bao, Y. , Wang, Y. Y. , Li, Z. , Zheng, X. , Long, S. , & Wang, Y. Y. (2019). Differences between generalized Q‐sampling imaging and diffusion tensor imaging in visualization of crossing neural fibers in the brain. Surgical and Radiologic Anatomy, 41(9), 1019–1028. 10.1007/s00276-019-02264-1 31144009 PMC6694094

[hbm26252-bib-0014] Kertesz, A. (2007). Western aphasia battery‐revised (WAB‐R). The Psychological Corporation.

[hbm26252-bib-0015] Keser, Z. , Meier, E. L. , Stockbridge, M. D. , Breining, B. L. , Sebastian, R. , & Hillis, A. E. (2021). Thalamic nuclei and thalamocortical pathways after left hemispheric stroke and their association with picture naming. Brain Connectivity, 11(7), 553–565. 10.1089/brain.2020.0831 33797954 PMC8558071

[hbm26252-bib-0016] Keser, Z. , Sebastian, R. , Hasan, K. M. , & Hillis, A. E. (2020). Right hemispheric homologous language pathways negatively predicts poststroke naming recovery. Stroke, 51(3), 1002–1005. 10.1161/STROKEAHA.119.028293 31884909 PMC7042036

[hbm26252-bib-0017] Kiran, S. , & Thompson, C. K. (2019). Neuroplasticity of language networks in aphasia: Advances, updates, and future challenges. Frontiers in Neurology, 10, 295. 10.3389/fneur.2019.00295 31001187 PMC6454116

[hbm26252-bib-0018] Klimovich‐Gray, A. , & Bozic, M. (2019). Domain‐general and domain‐specific computations in single word processing. NeuroImage, 202, 116112. 10.1016/j.neuroimage.2019.116112 31437552

[hbm26252-bib-0019] Kourtidou, E. , Kasselimis, D. , Angelopoulou, G. , Karavasilis, E. , Velonakis, G. , Kelekis, N. , Zalonis, I. , Evdokimidis, I. , Potagas, C. , & Petrides, M. (2021). The role of the right hemisphere white matter tracts in chronic aphasic patients after damage of the language tracts in the left hemisphere. Frontiers in Human Neuroscience, 15, 226. 10.3389/fnhum.2021.635750 PMC8258417 34239424

[hbm26252-bib-0020] Lam, J. M. C. , & Wodchis, W. P. (2010). The relationship of 60 disease diagnoses and 15 conditions to preference‐based health‐related quality of life in Ontario hospital‐based long‐term care residents. Medical Care, 48(4), 380–387. 10.1097/MLR.0b013e3181ca2647 20220536

[hbm26252-bib-0021] Marchina, S. , Zhu, L. L. , Norton, A. , Zipse, L. , Wan, C. Y. , & Schlaug, G. (2011). Impairment of speech production predicted by lesion load of the left arcuate fasciculus. Stroke, 42(8), 2251–2256. 10.1161/STROKEAHA.110.606103 21719773 PMC3167233

[hbm26252-bib-0022] O'Brien, K. R. , Kober, T. , Hagmann, P. , Maeder, P. , Marques, J. , Lazeyras, F. , Krueger, G. , & Roche, A. (2014). Robust T1‐weighted structural brain imaging and morphometry at 7T using MP2RAGE. PLoS One, 9(6), e99676. 10.1371/journal.pone.0099676 24932514 PMC4059664

[hbm26252-bib-0023] Pani, E. , Zheng, X. , Wang, J. , Norton, A. , & Schlaug, G. (2016). Right hemisphere structures predict poststroke speech fluency. Neurology, 86(17), 1574–1581. 10.1212/WNL.0000000000002613 27029627 PMC4844242

[hbm26252-bib-0024] Pedersen, P. M. , Stig Jørgensen, H. , Nakayama, H. , Raaschou, H. O. , & Olsen, T. S. (1995). Aphasia in acute stroke: Incidence, determinants, and recovery. Annals of Neurology, 38(4), 659–666. 10.1002/ana.410380416 7574464

[hbm26252-bib-0025] Sammler, D. , Kotz, S. A. , Eckstein, K. , Ott, D. V. M. , & Friederici, A. D. (2010). Prosody meets syntax: The role of the corpus callosum. Brain, 133(9), 2643–2655. 10.1093/brain/awq231 20802205

[hbm26252-bib-0026] Schilling, K. G. , Yeh, F. C. , Nath, V. , Hansen, C. , Williams, O. , Resnick, S. , Anderson, A. W. , & Landman, B. A. (2019). A fiber coherence index for quality control of B‐table orientation in diffusion MRI scans. Magnetic Resonance Imaging, 58, 82–89. 10.1016/j.mri.2019.01.018 30682379 PMC6401245

[hbm26252-bib-0027] Steinmann, S. , & Mulert, C. (2012). Functional relevance of interhemispheric fiber tracts in speech processing. Journal of Neurolinguistics, 25(1), 1–12. 10.1016/j.jneuroling.2011.07.003

[hbm26252-bib-0028] Stockert, A. , Wawrzyniak, M. , Klingbeil, J. , Wrede, K. , Kümmerer, D. , Hartwigsen, G. , Kaller, C. P. , Weiller, C. , & Saur, D. (2020). Dynamics of language reorganization after left temporo‐parietal and frontal stroke. Brain, 143(3), 844–861. 10.1093/brain/awaa023 32068789

[hbm26252-bib-0029] Swinburn, K. , Porter, G. , & Howard, D. (2005). Comprehensive aphasia test. Psychology Press.

[hbm26252-bib-0030] Veraart, J. , Novikov, D. S. , Christiaens, D. , Ades‐aron, B. , Sijbers, J. , & Fieremans, E. (2016). Denoising of diffusion MRI using random matrix theory. NeuroImage, 142, 394–406. 10.1016/j.neuroimage.2016.08.016 27523449 PMC5159209

[hbm26252-bib-0031] Wan, C. Y. , Zheng, X. , Marchina, S. , Norton, A. , & Schlaug, G. (2014). Intensive therapy induces contralateral white matter changes in chronic stroke patients with Broca's aphasia. Brain and Language, 136, 1–7. 10.1016/j.bandl.2014.03.011 25041868 PMC4425280

[hbm26252-bib-0032] Yeh, F. C. , Badre, D. , & Verstynen, T. (2016). Connectometry: A statistical approach harnessing the analytical potential of the local connectome. NeuroImage, 125, 162–171. 10.1016/j.neuroimage.2015.10.053 26499808

[hbm26252-bib-0033] Yeh, F. C. , Liu, L. , Hitchens, T. K. , & Wu, Y. L. (2017). Mapping immune cell infiltration using restricted diffusion MRI. Magnetic Resonance in Medicine, 77(2), 603–612. 10.1002/mrm.26143 26843524 PMC8052951

[hbm26252-bib-0034] Yeh, F. C. , Panesar, S. , Barrios, J. , Fernandes, D. , Abhinav, K. , Meola, A. , & Fernandez‐Miranda, J. C. (2019). Automatic removal of false connections in diffusion MRI tractography using topology‐informed pruning (TIP). Neurotherapeutics, 16(1), 52–58. 10.1007/s13311-018-0663-y 30218214 PMC6361061

[hbm26252-bib-0035] Yeh, F. C. , & Tseng, W. Y. I. (2011). NTU‐90: A high angular resolution brain atlas constructed by q‐space diffeomorphic reconstruction. NeuroImage, 58(1), 91–99. 10.1016/j.neuroimage.2011.06.021 21704171

[hbm26252-bib-0036] Yeh, F. C. , Verstynen, T. D. , Wang, Y. , Fernández‐Miranda, J. C. , & Tseng, W. Y. I. (2013). Deterministic diffusion fiber tracking improved by quantitative anisotropy. PLoS One, 8(11), e80713. 10.1371/journal.pone.0080713 24348913 PMC3858183

[hbm26252-bib-0037] Yeh, F. C. , Vettel, J. M. , Singh, A. , Poczos, B. , Grafton, S. T. , Erickson, K. I. , Tseng, W. Y. I. , & Verstynen, T. D. (2016). Quantifying differences and similarities in whole‐brain white matter architecture using local connectome fingerprints. PLoS Computational Biology, 12(11), e1005203. 10.1371/journal.pcbi.1005203 27846212 PMC5112901

[hbm26252-bib-0038] Yeh, F. C. , Wedeen, V. J. , & Tseng, W. Y. I. (2010). Generalized q‐sampling imaging. IEEE Transactions on Medical Imaging, 29(9), 1626–1635. 10.1109/TMI.2010.2045126 20304721

