# Supplementary material for: Right hemispheric structural connectivity and poststroke language recovery
Source: Hum Brain Mapp. 2023 Feb 28;44(7):2897–904. doi: 10.1002/hbm.26252 (PMC10089089; doi:10.1002/hbm.26252)
Supplement: Supplementary file 1 — Data S1: Supporting information [file HBM-44-2897-s001.docx]

SUPPLEMENTARY MATERIAL

Right hemispheric structural connectivity and post-stroke language recovery

Aleksi J. Sihvonen^1,2,3,4,5^, Veronika Vadinova^1,2,3^, Kimberley L. Garden^1,2,3^, Marcus Meinzer^6^, Tracy Roxbury^1^, Kate O’Brien^1^, David Copland^1,2,3^, Katie L. McMahon^7^, Sonia L.E. Brownsett^1,2,3^

1. Queensland Aphasia Research Centre, University of Queensland, Herston, Australia
2. School of Health and Rehabilitation Sciences, University of Queensland, Brisbane, Australia
3. Centre of Research Excellence in Aphasia Recovery and Rehabilitation, La Trobe University, Melbourne, Australia
4. Cognitive Brain Research Unit (CBRU), University of Helsinki, Helsinki, Finland
5. Centre of Excellence in Music, Mind, Body and Brain, University of Helsinki, Helsinki, Finland
6. Department of Neurology, University Medicine Greifswald, Greifswald, Germany
7. School of Clinical Sciences, Centre for Biomedical Technologies, Queensland University of Technology, Brisbane, Australia

Corresponding author:
Dr Aleksi Sihvonen MD, PhD
Queensland Aphasia Research Centre, University of Queensland, 296 Herston Road, Herston, QLD 4029, Australia
Email: a.sihvonen@uq.edu.au

Eight people with aphasia participated in an 8-week word-retrieval treatment delivered between the early subacute and chronic stage. Whilst a treated versus untreated comparison was planned, limitations on sample size due to COVID-19 restrictions rendered group comparisons underpowered. However, there was no difference between group scores across both behavioural scores and across all time points (p = 0.399−0.943). Descriptive behavioural data (i.e., age and language scores at both time points) are reported at the group-level and for individual participants (Supplementary Table 1).

**Supplementary Table 1.** Patient (grouped by group allocation) behavioural *t*-scores on the Comprehensive Aphasia Test, with age at start of study (years). CHR = Chronic stage; Comp = Speech comprehension *t*-score; ES = Early subacute stage; Prod = Speech production *t*-score.

| **Patient** | **Age** | **Comp_ES** | **Comp_CHR** | **Prod_ES** | **Prod_CHR** |
| --- | --- | --- | --- | --- | --- |
| 1 | 86 | 53 |  | 58 |  |
| 2 | 65 | 42 | 48 | 35 | 50 |
| 3 | 54 | 55 | 65 | 56 | 68 |
| 4 | 58 | 50 | 52 | 51 | 59 |
| 5 | 45 | 59 | 52 | 51 | 61 |
| 6 | 55 | 62 | 49 | 63 | 63 |
| 7 | 70 | 52 | 57 | 46 | 52 |
| 8 | 61 | 57 | 62 | 57 | 62 |
| **Treatment group mean** | **61.8** | **53.8** | **55** | **52.1** | **59.3** |
| 9 | 64 | 59 | 65 | 58 | 74 |
| 10 | 70 | 52 | 55 | 64 | 64 |
| 11 | 72 | 43 | 46 | 42 | 41 |
| 12 | 65 | 60 | 58 | 69 | 65 |
| 13 | 74 | 45 |  |  |  |
| 14 | 66 | 47 | 54 | 55 | 62 |
| 15 | 68 | 45 | 51 | 48 | 56 |
| 16 | 82 | 41 |  | 57 |  |
| 17 | 52 | 46 | 58 | 50 | 59 |
| 18 | 73 | 54 |  | 59 |  |
| 19 | 51 | 74 | 74 | 69 | 75 |
| 20 | 66 | 52 |  | 43 |  |
| 21 | 73 | 61 |  | 64 |  |
| 22 | 42 | 52 |  | 47 |  |
| **Usual care mean** | **65.6** | **53.5** | **57.6** | **55.5** | **62** |
| **Total Mean** | **64.2** | **53.6** | **56.4** | **54.3** | **60.7** |

To ensure that left hemisphere structural characteristics have been adequately accounted for, we repeated the main longitudinal analyses and evaluated which early subacute stage whole-brain local connectomes were associated with longitudinal change (chronic>early subacute) in comprehension and production *t*-scores (n=15). In addition to age and lesion volume, baseline performance was included as an additional covariate to avoid its possible confounding effects^1^.

Similar to the main analysis, greater longitudinal improvement in language comprehension *t*-scores was associated with greater QA in the corpus callosum (body, forceps major and tapetum) (Supplementary Figure 1A) (p_FDR_<0.0125). Additional significant positive associations between improvement in language comprehension and early subacute QA were observed in the left fornix and cerebellar tract. Negative associations were not observed.

In language production, paralleling the main findings, only significant negative associations were observed. These revealed greater longitudinal improvement in production *t*-scores was associated with lower QA in segments of right corticospinal tract and cingulum as well as the corpus callosum (body, forceps minor) (Supplementary Figure 1B) (p_FDR_<0.0125). Additional negative associations were observed between the left thalamic radiation and language production *t*-scores.

# References

1. Hope TMH, Friston K, Price CJ, Leff AP, Rotshtein P, Bowman H. Recovery after stroke: Not so proportional after all? *Brain*. 2019;142:15–22.


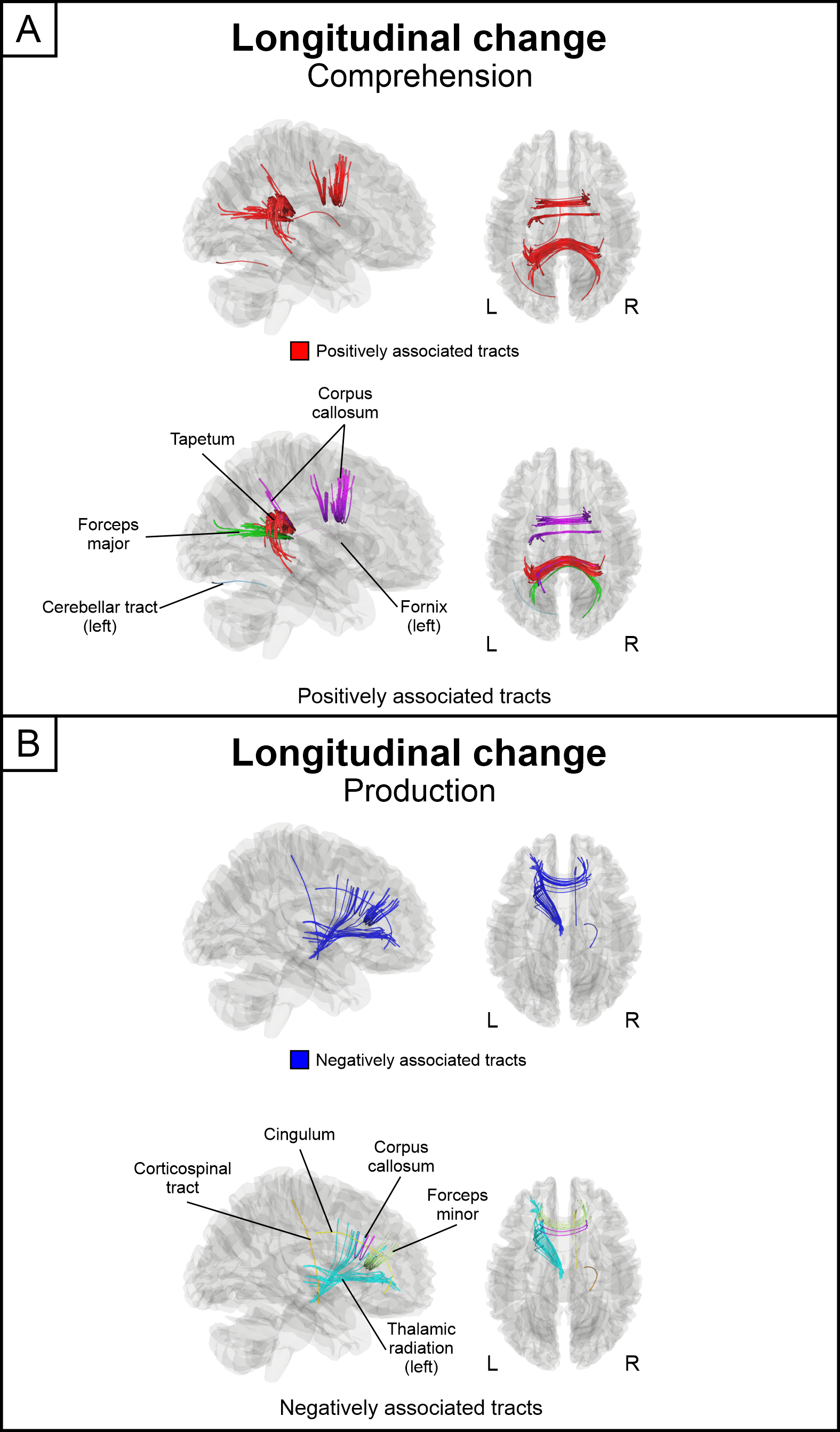


**Supplementary Figure 1.** Early subacute stage whole-brain connectomes associated with positive and negative longitudinal change in comprehension and production scores (A) Positively associated tracts with comprehension. (B) Negatively associated tracts with production. All results are thresholded at FDR < 0.0125 and adjusted for age, lesion volume and respective baseline language score, N = 15. L = left; R = Right.
